# The Translation and Adaptation of the Labour Force Survey Disability Module (LFS-DM) Into Hindi

**DOI:** 10.7759/cureus.76475

**Published:** 2024-12-27

**Authors:** Deepak Kumar, Richa Richa, Rajan Kumar, Himel Mondal, Khageshwar Kumar, Sanyogita Singh, Pratima Gupta, Saurabh Varshney

**Affiliations:** 1 Physical Medicine and Rehabilitation, All India Institute of Medical Sciences, Deoghar, IND; 2 Community and Family Medicine, All India Institute of Medical Sciences, Deoghar, IND; 3 Pediatrics, All India Institute of Medical Sciences, Deoghar, IND; 4 Physiology, All India Institute of Medical Sciences, Deoghar, IND; 5 Research, All India Institute of Medical Sciences, Deoghar, IND; 6 Microbiology, All India Institute of Medical Sciences, Deoghar, IND; 7 Otolaryngology, All India Institute of Medical Sciences, Deoghar, IND

**Keywords:** conceptual equivalence, cultural adaptation, disability assessment, disability module, healthcare tool, hindi translation, internal consistency, labour force survey, pilot testing, test-retest reliability

## Abstract

Background and objective

Disability assessments are crucial for identifying barriers faced by individuals with disabilities, particularly in countries like India, where disability is often underreported. The Labour Force Survey Disability Module (LFS-DM) is a widely used tool for disability assessment. It is available in English, and Indian Hindi-speaking people who are not proficient in English may face difficulties in responding to it. This study aimed to translate and culturally adapt the LFS-DM into Hindi. This will improve its accessibility and relevance for Hindi-speaking populations in India.

Methods

The LFS-DM was translated into Hindi using a forward and backward translation process involving three bilingual experts. The resulting Hindi version was reviewed for conceptual equivalence (on a 10-point scale) by three healthcare professionals. Pilot testing was conducted with 51 participants with various disabilities who completed the questionnaire and participated in cognitive interviews. The test-retest reliability was assessed using the intraclass correlation coefficient (ICC) with a two-week interval between administrations.

Results

The linguistic validation process ensured that the Hindi version of the LFS-DM was conceptually equivalent to the original English version with an average score of 9.57 ±0.48. Pilot testing revealed that all participants (27 males, 24 females, mean age: 45.6 ±7.8 years) easily understood the questions, with no major issues reported. Test-retest reliability was good to excellent, with ICC ranging from 0.843 (minimum) to 0.999 (maximum).

Conclusions

The Hindi-translated LFS-DM was linguistically valid and had similar conceptual equivalence with the original questionnaire. It showed good to excellent test-retest reliability. Hence, it can be used in the assessment of disability among Hindi-speaking populations. This tool can be used in future research, healthcare assessments, and policy development, contributing to more inclusive practices for persons with disabilities in India.

## Introduction

Assessing disability among individuals is vital to understanding their functional limitations, participation restrictions, and the impact of disabilities on their social and economic lives [1]. For persons with disabilities (PWDs), this process is essential for recognizing their needs and fostering societal inclusion in areas like employment, education, and healthcare [2]. Assessing disability in a structured, reliable manner enables the identification of systemic barriers and enhances efforts to ensure compliance with international commitments [3]. In multilingual and diverse societies like India, the reliance on English-language questionnaires for disability assessment poses significant challenges [4]. For patients, especially those from rural or non-English-speaking backgrounds, understanding and accurately responding to English survey questions can be daunting [5]. Many respondents may have limited proficiency in English, leading to confusion, misinterpretation, or even non-responses.

Local surveyors, particularly those not fluent in English, may also face hurdles. They may struggle with accurately translating technical terms or questions on the spot during data collection [6]. This can result in inconsistencies in responses, reduced data quality, and an overall lack of reliability in the findings. Additionally, surveyors may find it challenging to build rapport with respondents when using a language that feels foreign or inaccessible to both parties, further limiting the effectiveness of the data collection process [7]. 

The Labour Force Survey Disability Module (LFS-DM) was developed to be included in labor force surveys, but it may also be used in population-based surveys that collect data on employment [8]. This questionnaire is available in English. However, in India, the majority (about 43.63%) of the population speaks Hindi [9]. Hence, for using this questionnaire in an Indian context, it was necessary to translate and adapt it into Hindi. We believe it would enable better understanding and participation among respondents. It would also empower local surveyors to administer the survey with confidence and precision, thereby improving data collection efforts.

## Materials and methods

Study design and setting

This was a cross-sectional, descriptive study conducted at the All India Institute of Medical Sciences, Deoghar, Jharkhand, India. The study was approved by the Institutional Ethics Committee (approval number: 2023-93-EMP-03). The study involved the translation of the questionnaire and pilot testing the questionnaire. The participants for the pilot testing were recruited from the outpatient department of the hospital, ensuring a diverse representation of individuals with different types and levels of disabilities. The study was conducted from June 1, 2024, to November 30, 2024.

The questionnaire

The LFS-DM comprises five sections designed to address various aspects of disability. The first section focuses on disability identification (eight questions regarding vision, hearing, mobility, cognition, self-care, communication, anxiety, and depression). The second section assesses the barriers to employment and targets working-age individuals with disabilities who are not employed, exploring challenges preventing them from working (two questions). The third section applies to employed individuals with disabilities, identifying workplace adjustments needed for their roles (two questions). The fourth section examines attitudes related to employability and colleagues' willingness to work with persons with disabilities (two questions). The fifth section assesses access to social benefits among working-age individuals with disabilities, regardless of their employment status (three questions). Hence, this questionnaire has a total of 17 questions or statements with closed-ended response options. The questionnaire is available in Appendices.

Translation

**Figure 1:**
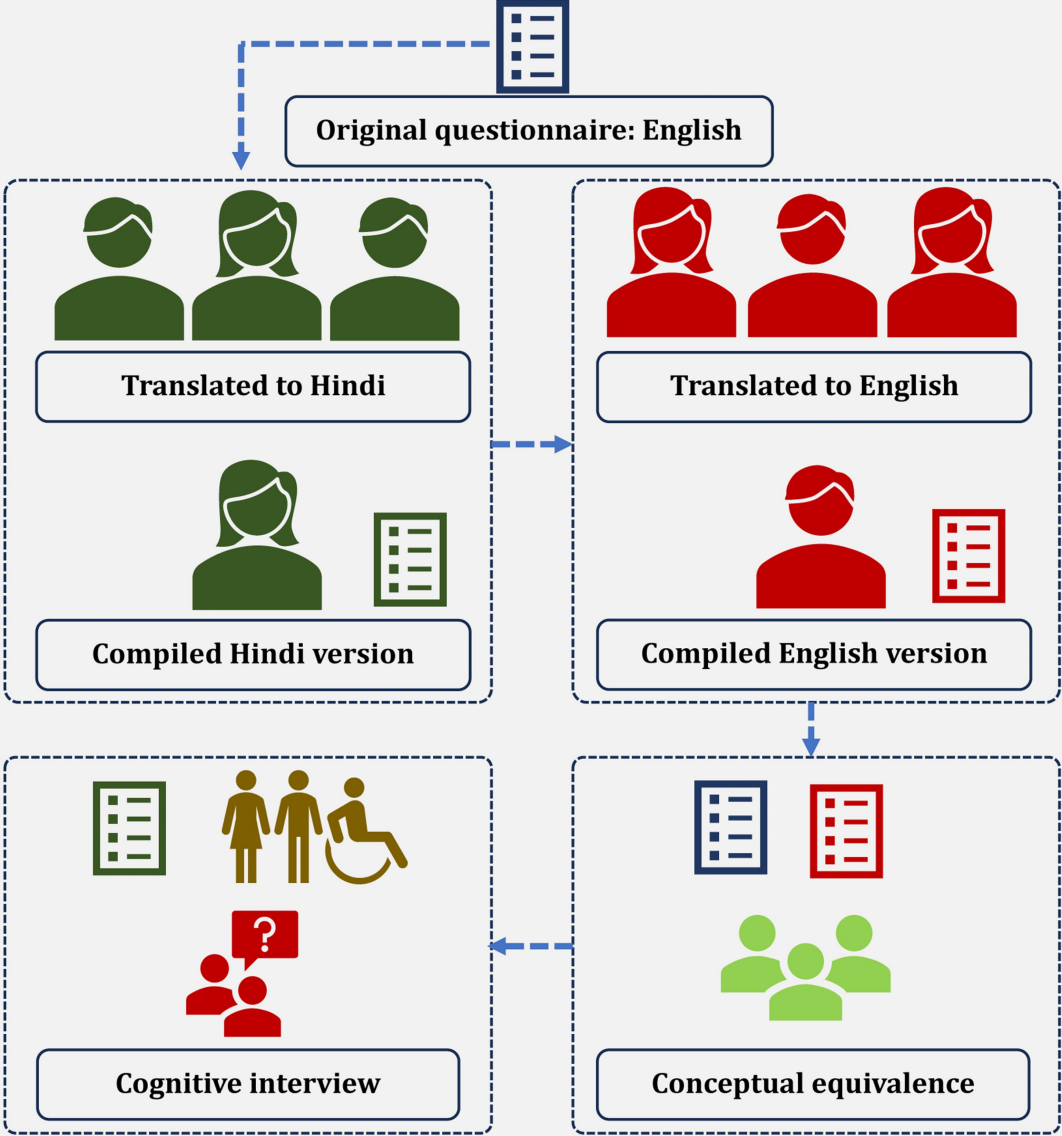
Brief summary of study process showing the forward translation, backward translation, conceptual equivalence check, and cognitive interview Source: This figure was prepared in Microsoft Word 2021 by Dr. Himel Mondal for this article

This study employed a structured process to translate LFS-MD into Hindi, ensuring linguistic and cultural equivalence while maintaining the integrity of the original questionnaire [10]. The study process is illustrated in Figure 1.

The questionnaire was initially translated from English to Hindi by three bilingual experts proficient in both languages and familiar with disability-related terminology. Their translations were reconciled by one of the authors to produce a unified version. Then, the Hindi version of the questionnaire was back-translated into English by three additional bilingual experts who were not involved in the initial translation process. The back-translated versions were reconciled by another author. 

The reconciled Hindi version was then reviewed by three expert doctors in physical medicine and rehabilitation to assess its conceptual equivalence between the back-translated English version and the original English version. This step ensured that the Hindi questionnaire accurately captured the meaning and intent of the original questions, particularly in the context of disability-related constructs. The expert rated each of the questions (i.e., item) on a 10-point visual analog scale to rate the equivalence to the original English version.

Pilot testing and cognitive interview 

The finalized Hindi questionnaire underwent pilot testing with 51 patients representing diverse backgrounds and disabilities. We included any patients aged 18 years or older who self-identified as having a disability, could understand and communicate in Hindi, and gave informed consent. The exclusion criteria included individuals with severe cognitive impairments or communication barriers that prevent them from comprehending the questions, those unwilling or unable to provide consent, and individuals whose disabilities do not align with the scope of the questionnaire (e.g., temporary disabilities). Each participant completed the questionnaire and participated in a cognitive interview to evaluate their understanding of the questions. During the interviews, they were asked to elaborate on their interpretation of the questions and highlight any difficulties faced in comprehension or responding [11]. They were again called for consultation after two weeks, and the same questionnaire was applied to get re-test responses.

The pilot testing revealed that all questions in the Hindi version were easily understood by the participants. The cognitive interviews confirmed that the Hindi questionnaire was clear, culturally relevant, and linguistically accessible, with no major issues reported.

Statistical analysis

Descriptive statistics were used to analyze the data collected during pilot testing. The demographic characteristics of the participants were summarized using numbers and percentages, means, and standard deviations (SD), as appropriate. For test and re-test comparison, the responses were coded with numerical values (e.g., very supportive = 1, somewhat supportive = 2, and not supportive = 3). The numbers were not based on positivity (e.g., very supportive = 3) as we aimed to compare the final rating only, which does not require any direction. Test-retest reliability was evaluated by the intraclass correlation coefficient (ICC). We used Microsoft Excel 2010 (Microsoft Corporation, Redmond, WA) for descriptive and IBM SPSS Statistics version 20 (IBM Corp., Armonk, NY) for inferential statistical tests. A p-value <0.05 was considered statistically significant.

## Results

A total of three experts forward-translated the questionnaire. One author (DK) compiled the Hindi translation into one final version. Another three experts back-translated it to English, and another author (HM) compiled it to make a final English version. This version was checked for conceptual equivalence by three doctors practicing PMR, and their conceptual equivalence score is shown in Table 1.

**Table 1:** Conceptual equivalence of back-translated English questionnaire and original English questionnaire The p-value is of ICC where the ratings of three observers were compared CI: confidence interval; ICC: intraclass correlation coefficient; SD: standard deviation

Rater	Average rating (n = 17) (mean ± SD)	ICC	96% CI (lower bound - upper bound)	P-value
First rater	9.41 ± 0.71	0.79	0.53 - 0.92	<0.0001
Second rater	9.65 ± 0.49			
Third rater	9.65 ± 0.49			
Average of three	9.57 ± 0.48			

Inter-rater agreement was satisfactory, and the score was high enough to conclude that the back-translated and original English versions were conceptually equivalent.

A total of 51 participants (27 males, 24 females, mean age: 45.6 ± 7.8 years) completed the pilot testing of the Hindi-translated LFS-DM. The demographic characteristics of the participants are shown in Table 2.

**Table 2:** Demographic characteristics of the participants in the pilot testing ^*^P-value of the binomial test. ^†^P-value of the chi-square test. A significant p-value indicates that the distribution of participants in categories did not occur by chance

Characteristic	Category	N (%)	P-value
Sex	Male	27 (52.94)	0.78^*^
	Female	24 (47.06)	
Marital status	Married	49 (96.08)	<0.0001^*^
	Unmarried	2 (3.92)	
	Widow/Widower/Divorced	0	
Education	<5th standard	30 (58.82)	<0.0001^†^
	5th - 10th standard	10 (19.61)	
	>10th - 12th standard	7 (13.73)	
	>12 standard - graduation	4 (7.84)	
Residence	Rural	48 (94.12)	<0.0001^*^
	Urban	3 (5.88)	
Socioeconomic status	Upper class	0	<0.0001^†^
	Upper middle class	0	
	Lower middle class	4 (7.84)	
	Upper lower class	13 (25.49)	
	Lower class	34 (66.67)	
Language (can read)	Hindi	42 (82.35)	<0.0001^*^
	English	9 (17.65)	
Language (can write)	Hindi	42 (82.35)	<0.0001^*^
	English	9 (17.65)	

Most participants were married, and there were no widows, widowers, or divorced individuals in the cohort. A significant portion of the participants were illiterate, while others had varying levels of education, including a small number with secondary and higher education qualifications. Socioeconomically, the participants were primarily from the lower and upper lower classes. Regarding literacy, most participants could read and write in Hindi, with a few able to do so in English.

During pilot testing, all participants easily understood the questionnaire, and the cognitive interviews confirmed clarity and comprehension. Test and re-test scores are shown in Table 3.

**Table 3:** Test and re-test score of the items and their correlation CI: confidence interval; ICC: intraclass correlation coefficient; SD: standard deviation

Item number	Test (n = 51)	Re-test (n = 51)	Statistical test parameters		
	Mean ± SD		ICC	95% CI (lower bound - upper bound)	P-value
1	1.71 ± 0.81	1.63 ± 072	0.967	0.943 - 0.981	<0.0001
2	1.08 ± 0.27	1.08 ± 0.27	0.843	0.725 - 0.91	<0.0001
3	2.37 ± 1	2.19 ± 0.92	0.98	0.964 - 0.988	<0.0001
4	1.45 ± 0.67	1.43 ± 0.61	0.962	0.934 - 0.979	<0.0001
5	2.20 ± 1	2.16 ± 1.05	0.961	0.931 - 0.978	<0.0001
6	1.33 ± 0.95	1.33 ± 0.86	0.988	0.979 - 0.993	<0.0001
7	2.59 ± 2.51	2.57 ± 2.07	0.997	0.994 - 0.998	<0.0001
8	2.9 ± 2.46	2.88 ± 2.45	0.998	0.996 - 0.999	<0.0001
9	8.27 ± 1.86	8.29 ± 1.81	0.999	0.997 - 0.999	<0.0001
10	2.47 ± 1.6	2.51 ± 1.6	0.996	0.993 - 0.998	<0.0001
11	2.41 ± 1.53	2.37 ± 1.5	0.996	0.993 - 0.998	<0.0001
12	2.94 ± 1.79	2.98 ± 1.85	0.997	0.995 - 0.998	<0.0001
13	3.1 ± 1.54	3.16 ± 1.5	0.994	0.989 - 0.996	<0.0001
14	3.04 ± 1.62	2.96 ± 1.56	0.989	0.98 - 0.994	<0.0001
15	2.29 ± 0.7	2.33 ± 0.71	0.98	0.966 - 0.989	<0.0001
16	1.98 ± 0.32	2.02 ± 0.37	0.913	0.847 - 0.95	<0.0001
17	2.03 ± 0.4	1.96 ± 0.34	0.877	0.785 - 0.93	<0.0001

Test-retest reliability showed good (0.7 - 0.9) to excellent (>0.9) reliability, with ICC ranging from 0.843 (minimum) to 0.999 (maximum), indicating strong consistency over time.

## Discussion

This study translated and adapted the LFS-DM into Hindi, ensuring its reliability for use in Hindi-speaking populations. The process involved careful linguistic validation, including forward and backward translation, followed by expert review to ensure conceptual equivalence. The results from the pilot testing, which involved 51 participants with diverse disabilities, showed that the Hindi version of the questionnaire was well understood by respondents, and no significant issues related to comprehension or clarity were identified.

The employment rates for people with disabilities in India are very low, according to the National Centre for Promotion of Employment for Disabled People. Employment is only 0.54% in the public sector, 0.28% in the private sector, and 0.05% in multinational corporations. The economic prosperity of India is seriously hampered by this poor representation [12]. Many people with disabilities want to work but face significant barriers, leading to low employment rates [13]. Job placement and inclusion of persons with disabilities in India remain a major challenge. Despite laws and policies to protect their rights, many barriers still prevent their full participation in the workforce [14]. Many areas of India are not surveyed due to multiple hurdles. In addition, disability questions answered by household heads often lead to misreporting, especially among wealthier households, due to stigma, societal negativity, and fears of losing power [15]. Hence, it is high time to survey with local surveyors without any language barrier.

The Hindi adaptation of the questionnaire can play a crucial role in assessing the labor force participation of persons with disabilities in India. It can help identify employment rates, barriers to workforce entry, and the impact of workplace accessibility and accommodations [16]. The data generated can guide policymakers in evaluating existing initiatives and implementing targeted interventions to enhance job opportunities for PWDs. If any researcher would like to use the questionnaire for research purposes, they can get a printable version of the questionnaire from the first author of this article via email (deepak.pmr@aiimsdeoghar.edu.in).

Strengths and limitations

One of the key strengths of this study is its attention to cultural relevance. Disability in India is often accompanied by diverse cultural perceptions and stigma [17], and the translation process considered these aspects to ensure the questionnaire’s items were not only linguistically accurate but also culturally appropriate. This is particularly important in a country like India, where disability is perceived differently across regions, communities, and social strata [18].

The study has some limitations. The sample for the cognitive interview was a convenience sample from a tertiary care hospital in a particular state in India, and it may not fully represent the diversity of the Indian population, particularly in terms of socioeconomic status or geographic variation. Future research with a larger, more diverse sample will be necessary to further validate the questionnaire's applicability across different populations.

## Conclusions

The translation and adaptation of the LFS-DM into Hindi is a step toward improving the accessibility and cultural relevance of disability data collection in India. By ensuring linguistic clarity and conceptual equivalence, the adapted module provides a reliable tool for capturing the experiences of Hindi-speaking individuals with disabilities. The Hindi version of the questionnaire demonstrated good to excellent test-retest reliability, making it a reliable tool for assessing disability-related issues in Hindi-speaking populations. Pilot testing and cognitive interviews confirmed that the questionnaire was easily understood by participants, and the cultural adaptation process ensured that the tool was both contextually and linguistically appropriate for the target population. The adapted Hindi module will help stakeholders in disability data collection, supporting inclusive labor policies, and evidence-based decision-making.

## Appendices

**Table 4:** Questions and response option of Labour Force Survey Disability Module The number before the response options indicate code of the response

Item number	Question	Response options
1	Do you have difficulty seeing, even if wearing glasses?	1. No difficulty, 2. Some difficulty, 3. A lot of difficulty, 4. Cannot do at all, 8. Refused, 9. Don’t know
2	Do you have difficulty hearing, even if using a hearing aid(s)?	1. No difficulty, 2. Some difficulty, 3. A lot of difficulty, 4. Cannot do at all, 8. Refused, 9. Don’t know
3	Do you have difficulty walking or climbing steps?	1. No difficulty, 2. Some difficulty, 3. A lot of difficulty, 4. Cannot do at all, 8. Refused, 9. Don’t know
4	Do you have difficulty remembering or concentrating?	1. No difficulty, 2. Some difficulty, 3. A lot of difficulty, 4. Cannot do at all, 8. Refused, 9. Don’t know
5	Do you have difficulty with self-care, such as washing all over or dressing?	1. No difficulty, 2. Some difficulty, 3. A lot of difficulty, 4. Cannot do at all, 8. Refused, 9. Don’t know
6	Using your usual language, do you have difficulty communicating, for example understanding or being understood?	1. No difficulty, 2. Some difficulty, 3. A lot of difficulty, 4. Cannot do at all, 8. Refused, 9. Don’t know
7	How often do you feel very anxious, nervous or worried?	1. Never, 2. A few times a year, 3. Monthly, 4. Weekly, 5. Daily, 8. Refused, 9. Don’t know
8	How often do you feel very sad or depressed?	1. Never, 2. A few times a year, 3. Monthly, 4. Weekly, 5. Daily, 8. Refused, 9. Don’t know
9	Which of the following factors would make it more likely for you to seek or find a job?	1. Getting higher qualifications/training/skills, 2. Availability of suitable transportation to and from workplace, 3. Help in locating appropriate jobs, 4. More positive attitudes towards persons with disabilities, 5. Availability of special equipment or assistive devices, 6. Availability of more flexible work schedules or work task arrangements, 7. Availability of a more accommodating workplace, 8. Other: Please specify ______, 98. Refused, 99. Don’t know
10	How supportive would your family members be if you decide to work?	1. Very supportive, 2. Somewhat supportive, 3. Not supportive, 8. Refused, 9. Don’t Know
11	Is your work schedule or work tasks arranged to account for difficulties you have in doing certain activities?	1. Yes, fully, 2. Yes, partially, 3. Not at all, 4. I do not have difficulties that require accommodation, 8. Refused, 9. Don’t Know
12	Has your workplace been modified to account for difficulties you have in doing certain activities?	1. Yes, fully, 2. Yes, partially, 3. Not at all, 4. I do not have difficulties that require accommodation, 8. Refused, 9. Don’t Know
13	In your view, how willing are employers to hire persons with disabilities?	1. Very willing, 2. Somewhat willing, 3. Unwilling, 8. Refused, 9. Don’t Know
14	In your view, how willing are workers to work alongside persons with disabilities?	1. Very willing, 2. Somewhat willing, 3. Unwilling, 8. Refused, 9. Don’t Know
15	Have the difficulties you have been officially recognized (certified) as a disability?	1. Yes, go to the next question, 2. No, end interview, 8. Refused, end interview, 9. Don’t know, end interview
16	Do you receive any cash benefits from the government linked to your disability?	1. Yes, 2. No, 8. Refused, 9. Don’t know
17	Do you receive any goods or services from the government linked to your disability?	1. Yes, 2. No, 8. Refused, 9. Don’t know
